# Recombinant platelet‐derived growth factor‐BB alleviates osteoarthritis in a rat model by decreasing chondrocyte apoptosis in vitro and in vivo

**DOI:** 10.1111/jcmm.16779

**Published:** 2021-07-11

**Authors:** Pengfei Zhu, Zhengchao Wang, Zhenxing Sun, Bokai Liao, Yu Cai

**Affiliations:** ^1^ Department of Cardiology Wuhan Fourth Hospital Puai Hospital Tongji Medical College Huazhong University of Science and Technology Wuhan China; ^2^ Department of Orthopedics Tongji Hospital Tongji Medical College Huazhong University of Science and Technology Wuhan China; ^3^ Department of Ultrasound Union Hospital Tongji Medical College Huazhong University of Science and Technology Wuhan China; ^4^ School of Chemistry and Chemical Engineering Guangzhou University Guangzhou China; ^5^ Department of Rehabilitation Wuhan Fourth Hospital Puai Hospital Tongji Medical College Huazhong University of Science and Technology Wuhan China

**Keywords:** apoptosis, knee, osteoarthritis, platelet‐derived growth factor

## Abstract

Osteoarthritis (OA) is a common joint disease that mainly affects the diarthrodial joints. Treatments for OA include non‐pharmacological interventions, topical and oral therapies, intra‐articular therapies and joint surgery. However, all the treatments mentioned above mainly aim to control the symptoms instead of improving or reversing the joint condition. In this research, we observed the effect of recombinant platelet‐derived growth factor (PDGF)‐BB on OA in a monosodium iodoacetate (MIA)–induced rat model and revealed the possible mechanisms. In vitro, the level of inflammation in the chondrocytes was gradually alleviated, and the apoptosis rate was gradually decreased by PDGF‐BB at increasing concentrations. The levels of p‐p38, Bax and caspase‐3 decreased, and the level of p‐Erk increased with increasing PDGF‐BB concentration. In vivo, PDGF‐BB could significantly reverse chondrocyte and matrix loss. Furthermore, high concentrations of PDGF‐BB could alleviate cartilage hyperplasia to remodel the tissue. The level of collagen II was up‐regulated, and the levels of collagen X and apoptosis were down‐regulated by increasing concentrations of PDGF‐BB. In conclusion, recombinant PDGF‐BB alleviated OA by down‐regulating caspase‐3‐dependent apoptosis. The effects of PDGF‐BB on OA mainly include inhibiting chondrocyte loss, reducing cartilage hyperplasia and osteophyte formation, and regulating collagen anabolism.

## 1 INTRODUCTION

Osteoarthritis (OA), defined as ‘a group of overlapping distinct entities with similar biologic, morphologic and clinical outcomes’,^1^ is the most challenging and common joint disease that mainly affects the diarthrodial joints. This condition can affect all tissues of the diarthrodial joint, including the articular cartilage, subchondral bone, ligaments, capsule and synovial membrane, ultimately leading to joint failure.^1^ Treatments for OA include non‐pharmacological interventions, such as exercise programmes and weight loss^2^, ^3^; topical and oral therapies, such as non‐steroidal anti‐inflammatory drugs, paracetamol and chondroitin sulphate products^4^, ^5^; intra‐articular therapies, such as corticosteroids and hyaluronan^6^; and joint surgery.^1^ However, all of the treatments mentioned above mainly aim to control the symptoms instead of improving or reversing the joint condition.

Apoptosis is a highly regulated and active process of cell death that is critical for maintaining homeostasis in the body. However, excessive apoptosis has negative effects due to abnormal cell loss. As a result, maintaining the balance between proliferation and cell death is important. Electron microscopy of normal and osteoarthritic cartilage showed that apoptosis was positively correlated with the severity of cartilage degradation and matrix loss. Recent studies have also shown that decreasing apoptosis of chondrocytes by aucubin, quercetin, tanshinone and microRNAs could inhibit OA.^7^, ^8^, ^9^, ^10^ Therefore, targeting apoptosis in chondrocytes could be an effective management strategy for OA.

Platelet‐derived growth factor (PDGF)‐BB, as a member of the PDGF cytokine family, plays an important role in wound healing via its effects on mitosis, chemotaxis and angiogenesis.^11^ This molecule is produced by osteoblasts, platelets and monocytes/macrophages and can easily interact with all three PDGF receptors.^12^ Moreover, PDGF‐BB was found to be an apoptosis inhibitor via the Erk1/2 and p38 pathways and exhibited cytoprotective effects.^13^, ^14^, ^15^ PDGF‐BB could suppress cartilage degradation and improve the anabolism of chondrocytes to alleviate OA in vivo.^16^, ^17^ Thus, PDGF‐BB could be a possible treatment for OA.

In this research, we mainly focused on the effects of recombinant PDGF‐BB on OA and revealed the possible mechanisms both in vitro and in vivo.

## 2 MATERIALS AND METHODS

### 2.1 Chondrocyte isolation and culture

Seven‐day‐old neonatal male Sprague‐Dawley rats were purchased from the Experimental Animal Center, Tongji Medical College, Huazhong University of Science and Technology. All animals were killed under deep anaesthesia [100 mg/kg chloral hydrate intraperitoneally (i.p.)]. After aseptic exposure of the knee articular cavity, pieces of articular cartilage were dissected and separated from the underlying bone and connective tissues. The cartilage was then cut into 1 × 1×1 mm^3^ pieces and washed three times with PBS. After 4‐6 hours of digestion with 1 g/L collagenase type II at 37℃, the suspension was centrifuged at 1000 rpm for 5 minutes to collect chondrocytes. The extracted chondrocytes were cultured in DMEM/F‐12 with 10% foetal calf serum and 1% penicillin and streptomycin at a density of 1 × 10^5^ cells/mL (1 × 10^5^ cells/well) and incubated in a humidified atmosphere of 5% CO_2_ at 37℃. The culture medium was changed every 2‐3 days, and the cells were passaged using a 0.25% trypsin‐EDTA solution when 80‐90% confluence had been attained. Only the second or third generation of cells was used in the subsequent experiments.

### 2.2 Induction of inflammation and PDGF‐BB processing

Recombinant rat PDGF‐BB was purchased from R&D Systems and reconstituted and stored following the instructions. Monosodium iodoacetate (MIA) was purchased from Aladdin, dissolved in normal saline and stored following the instructions. Chondrocytes were cultured in DMEM/F‐12 at a density of 1 × 10^5^ cells/mL in 6‐well plates and incubated with PDGF‐BB (0, 10, 50 or 100 ng/mL) pre‐treatment for 1 hour prior to 5 μM MIA stimulation for 24 hours.

### 2.3 Cell viability assay

Recombinant rat PDGF‐BB was purchased from R&D Systems and reconstituted and stored following the instructions. MIA was purchased from Aladdin, dissolved in normal saline and stored following the instructions. After 1 hour of incubation with MIA, chondrocytes (1 × 10^5^ cells/well) were cultured in 96‐well plates and treated with different concentrations of PDGF‐BB (0, 10, 50 or 100 ng/mL) for 24 hours. Cell viability was determined using a CCK‐8 kit according to the manufacturer's instructions. The absorbance at 450 nm was measured with a microplate reader (Leica Microsystems). All experiments were performed in triplicate.

### 2.4 Griess reaction and enzyme‐linked immunosorbent assays (ELISAs)

The level of NO in the culture medium was detected via the Griess reaction as previously described.^18^ The level of PGE2 in the culture medium was detected using ELISA kits (R&D Systems) following the manufacturer's instructions. All experiments were performed in triplicate.

### 2.5 Detection of apoptosis and the cell cycle by flow cytometry

Flow cytometry was used to confirm apoptosis and assess the cell cycle. MIA‐induced chondrocytes were incubated with different concentrations of PDGF‐BB for 24 hours. Then, the cells were digested in trypsin without EDTA and washed twice in PBS.

For the detection of apoptosis, approximately 1 × 10^5^ cells were resuspended in binding buffer, which included 2 μL of 50 μg/mL propidium iodide (PI) and 2 μL of 20 μg/mL Annexin V‐FITC. Following one hour of cell staining, flow cytometric acquisition was performed using a flow cytometer (BD). At least 10 000 cells were collected for each sample with a flow rate of 250 to 300 cells/s, and 488 nm laser excitation was used. Cells were considered apoptotic when they were negative for PI and positive for Annexin V‐FITC. The analyses were performed using ModFit LT software (BD).

For cell cycle assessment, cells were fixed with 70% ethanol overnight at 4℃. Subsequently, after ethanol elimination via centrifugation, the cells were washed twice in PBS. Then, the cells were suspended in PBS and incubated in RNase (10 μg/mL) for 30 minutes at 37℃. After PI staining for 5 minutes, the cell cycle was evaluated using a FACSCalibur (BD Immunocytometry Systems). The analyses were performed using FlowJo software (Tree Star, Inc)

### 2.6 Protein extraction and Western blot analysis

Total protein from cultured chondrocytes was extracted with a Total Protein Extraction Kit (Aspen, as1004). Protein concentration was measured using a BCA protein assay kit. Equal quantities of 40 μg of total protein were separated on 10% SDS‐PAGE gels and then transferred onto PVDF membranes. The membranes were incubated in blocking buffer (5% skim milk in TBS‐T) at room temperature for 2 hours and washed three times with TBS‐T for 5 minutes. The blocked membranes were then incubated with primary antibodies against Bax (1:2000, rabbit, CST), Bcl‐2 (1:1000, rabbit, Abcam), caspase‐3 (1:500, rabbit, Abcam), Erk1/2 (1:2000, CST, Abcam), p‐Erk1/2 (1:1000, rabbit, CST), p38 (1:2000, rabbit, CST), p‐p38 (1:500, rabbit, CST) and GAPDH (1:10 000, rabbit, Abcam) overnight at 4℃ and washed three times with TBS‐T for 5 minutes, followed by incubation with the appropriate secondary antibody (1:10 000, goat, Aspen) at room temperature for 2 hours. After TBS‐T washes, the target bands were finally developed using an enhanced chemiluminescence (ECL) kit and semiquantitatively analysed using densitometric methods.

### 2.7 Immunofluorescence staining

Chondrocyte climbing sheets were fixed with 3% (w/v) paraformaldehyde at room temperature for 5 minutes. After two washes with PBS, the climbing sheets were permeabilized with PBS buffer containing 0.5% Triton X‐100 for 20 minutes and blocked with 5% bovine serum albumin (BSA) in PBS buffer for 10 minutes. The climbing sheets were incubated with primary antibodies [Bax (1:1000, rabbit, CST), Bcl‐2 (1:1000, rabbit, Abcam), caspase‐3 (1:1000, rabbit, Abcam), Erk1/2 (1:1000, CST, Abcam), p‐Erk1/2 (1:1000, rabbit, CST), p38 (1:1000, rabbit, CST) and p‐p38 (1:1000, rabbit, CST)] for 30 minutes at 37℃ and washed three times with PBS. The climbing sheets were incubated with DyLight 594–conjugated goat anti‐rabbit IgG antibodies (1:500) at 37℃ for 30 minutes and washed three times with PBS buffer. After nuclear staining with DAPI, the climbing sheets were covered with buffered glycerine, and images were captured using fluorescence microscopy. Fluorescence intensity was measured using ImageJ software 2.1.

### 2.8 MIA‐induced OA model and PDGF‐BB intervention

Twelve‐week‐old Sprague‐Dawley rats weighing 300‐350 g were purchased from the Experimental Animal Center, Tongji Medical College, Huazhong University of Science and Technology. Animals were maintained in accordance with the Guide for the Care and Use of Laboratory Animals of the National Institutes of Health and were approved by the Ethics Committee for Animal Experimentation of Puai Hospital Affiliated with Huazhong University of Science and Technology. The MIA‐induced rat model of OA is shown in Figure 7A. A volume of 50 μL of MIA (with a concentration of 6 g/L) was injected into the left articular cavity, as shown in Figure 7A. After 3 weeks, X‐ray detection was performed to confirm the MIA‐induced OA model (Figure 7A). Then, the animals were divided into 4 groups: the control group (saline), the MIA+100 ng/mL PDGF‐BB group, the MIA+50 ng/mL PDGF‐BB group and the MIA+10 ng/mL PDGF‐BB group. Fifty microlitres of saline or PDGF‐BB was injected intra‐articularly once a week for 4 weeks. At 8 weeks, all animals were killed using an overdose of 10% urethane, and the knee joints were dissected for histopathology.

### 2.9 Histological analysis

The rat knee joint samples were fixed in 4% paraformaldehyde for 24 hours and then decalcified in 10% EDTA for 4 weeks. After paraffin embedding, 4‐μm‐thick sections were obtained for histological analysis. Haematoxylin and eosin (HE) staining and toluidine blue staining were carried out. The severity of the OA lesions was graded according to the Osteoarthritis Research Society International (OARSI) scoring system. Three observers who were blinded to the grouping quantified the score, and 3 scores were averaged.

### 2.10 Immunohistochemistry

The collagen II and collagen X levels in cartilage tissue were assessed using immunohistochemistry. After incubation at 60℃ for 1 hour, tissue sections were dewaxed in conventional xylene and rehydrated in gradient alcohol. The sections were incubated in 30% H_2_O_2_ for 15 minutes to block endogenous peroxidase activity and then incubated in 10% normal sheep serum to block non‐specific sites. Then, the sections were incubated with anti‐collagen II antibody (1:500, rabbit, CST) or anti‐collagen X antibody (1:300, rabbit, CST) overnight at 4℃. The sections were incubated with horseradish peroxidase (HRP)–labelled goat anti‐rabbit IgG (1:500) secondary antibody at room temperature in the dark for 1 hour. Colour development was performed by the DAB system. Immunostaining analysis was evaluated by measuring the ratio (%) of positive cells to the total number in the cartilage using Optimas 6.5 software.

### 2.11 Detection of apoptosis by TUNEL staining

Apoptosis in tissue sections was detected using a TUNEL kit (Roche) according to the instructions. Briefly, tissue sections were incubated with xylene for 40 minutes, absolute ethyl alcohol for 20 minutes, 95% alcohol for 10 minutes, 80% alcohol for 5 minutes and 70% alcohol for 5 minutes. After the sections were washed with distilled water, they were incubated with protease K for 15 minutes and then washed with PBS. Then, the sections were incubated with DAPI dye (Aspen) for 5 minutes at room temperature in the dark, and a fluorescent quencher (Aspen) was added. The images of stained tissues were captured by fluorescence microscopy, and the results were analysed using Optimas 6.5 software.

### 2.12 Data analysis

Statistical analyses were performed using SPSS 21.0. The normality of distribution was tested by a Q‐Q plot. The data were analysed using repeated measures ANOVA. A post hoc test of the p‐value was performed with the Bonferroni correction. A value of *P* < .05 was considered statistically significant.

## 3 RESULTS

### 3.1 Effect of PDGF‐BB on cell viability

**FIGURE 1:**
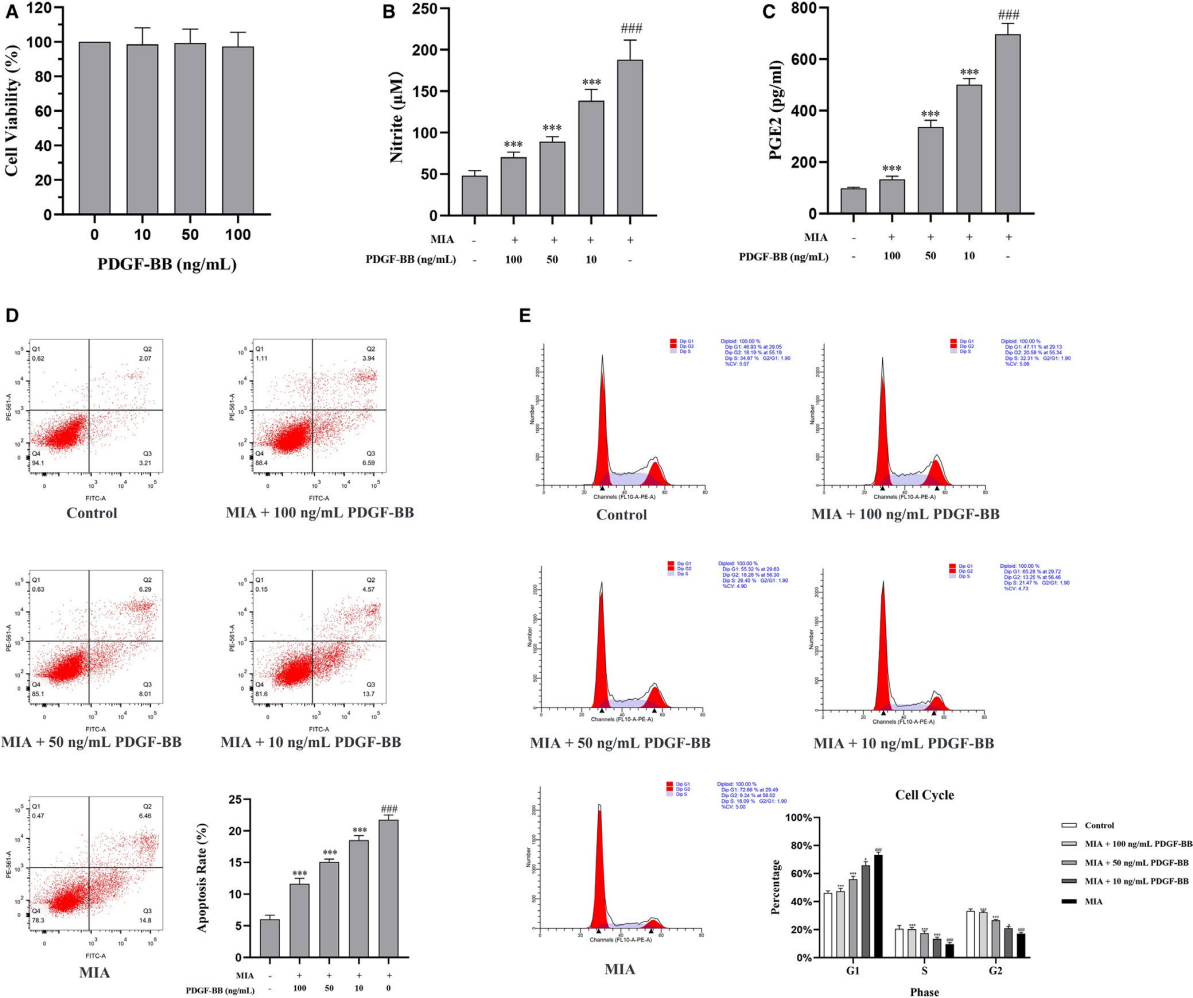
The effects of MIA and PDGF‐BB on chondrocytes. A, The effects of PDGF‐BB on normal chondrocytes were assessed by CCK‐8 assays. PDGF‐BB (10, 50 and 100 ng/mL) showed no effects on cell viability. B, The level of nitrite in the culture medium was assessed by the Griess reaction. MIA increased the level of nitrite in the culture medium. The increased nitrite was decreased by PDGF‐BB. C, The level of PGE2 in the culture medium was assessed by ELISAs. MIA increased the level of PGE2 in the culture medium. The increased PGE2 was decreased by PDGF‐BB. D, The cell cycle of chondrocytes was detected by flow cytometry. MIA induced G0/G1 arrest in chondrocytes. The induced G0/G1 arrest was alleviated by PFGF‐BB. E, The rate of apoptosis was detected by flow cytometry. MIA increased the apoptosis rate of chondrocytes. The increased apoptosis rate was decreased by PDGF‐BB. *, *P* < .05 vs. the MIA group; **, *P* < .01 vs. the MIA group; ***, *P* < .001 vs. the MIA group; and ###, *P* < .001 vs. the control group

Chondrocytes were incubated with different concentrations of PDGF‐BB (0, 10, 50 and 100 ng/mL) for 24 hours. Then, a CCK‐8 assay was used to measure cell viability. The data, as shown in Figure 1A, indicated that PDGF‐BB at this concentration range did not significantly affect cell viability.

### 3.2 PDGF‐BB weakens the inflammation induced by MIA

The levels of NO and PGE2 were measured in the MIA‐induced chondrocytes and after incubation with different concentrations of PDGF‐BB. As shown in Figure 1B,C, the NO and PGE2 levels were significantly increased in the MIA‐induced chondrocytes. After incubation with PDGF‐BB, the levels of NO and PGE2 decreased in a concentration‐dependent manner. These results indicated that PDGF‐BB could weaken the secretion of NO and PGE2 in chondrocytes after MIA induction.

### 3.3 PDGF‐BB ameliorates MIA‐induced chondrocyte cell cycle arrest in the G0/G1 phase

We detected the influence of PDGF‐BB on the cell cycle of the MIA‐induced chondrocytes by flow cytometry and found that PDGF‐BB intervention affected the cell cycle of the MIA‐induced chondrocytes. The cell cycle percentages for the G0/G1 phase were higher in the MIA group than in the control group (Figure 1E), while the cell cycle percentages for the S and G2 phases were lower in the MIA group than in the control group (Figure 1E). These results indicated that MIA induction prevented chondrocytes from transitioning from G0/G1 phase to S and G2 phases and thus resulted in more cells remaining in G0/G1 phase. After PDGF‐BB intervention, there was a change in the proportions in different cell cycle phases in the PDGF‐BB groups, as shown in Figure 1E. We observed decreased percentages in G0/G1 phase and increased percentages in S and G2 phases with an increase in PDGF‐BB concentration, indicating a more regular cell cycle for chondrocyte proliferation.

### 3.4 PDGF‐BB inhibits cell apoptosis in the MIA‐induced chondrocytes

Next, the apoptosis of the MIA‐induced chondrocytes was measured by flow cytometry. As shown in Figure 1D, the MIA‐induced chondrocytes displayed an increased apoptosis rate compared with the normal chondrocytes. However, the apoptosis rate of the MIA‐induced chondrocytes decreased after treatment with PDGF‐BB in a concentration‐dependent manner (Figure 1D). The inhibition rate of the MIA‐induced chondrocytes in the 100 ng/mL PDGF‐BB group was greater than that in the 50 ng/mL group and the 10 ng/mL group.

### 3.5 PFGF‐BB attenuates the expression of apoptosis‐related factors, down‐regulates p‐Erk and up‐regulates p‐p38

**FIGURE 2:**
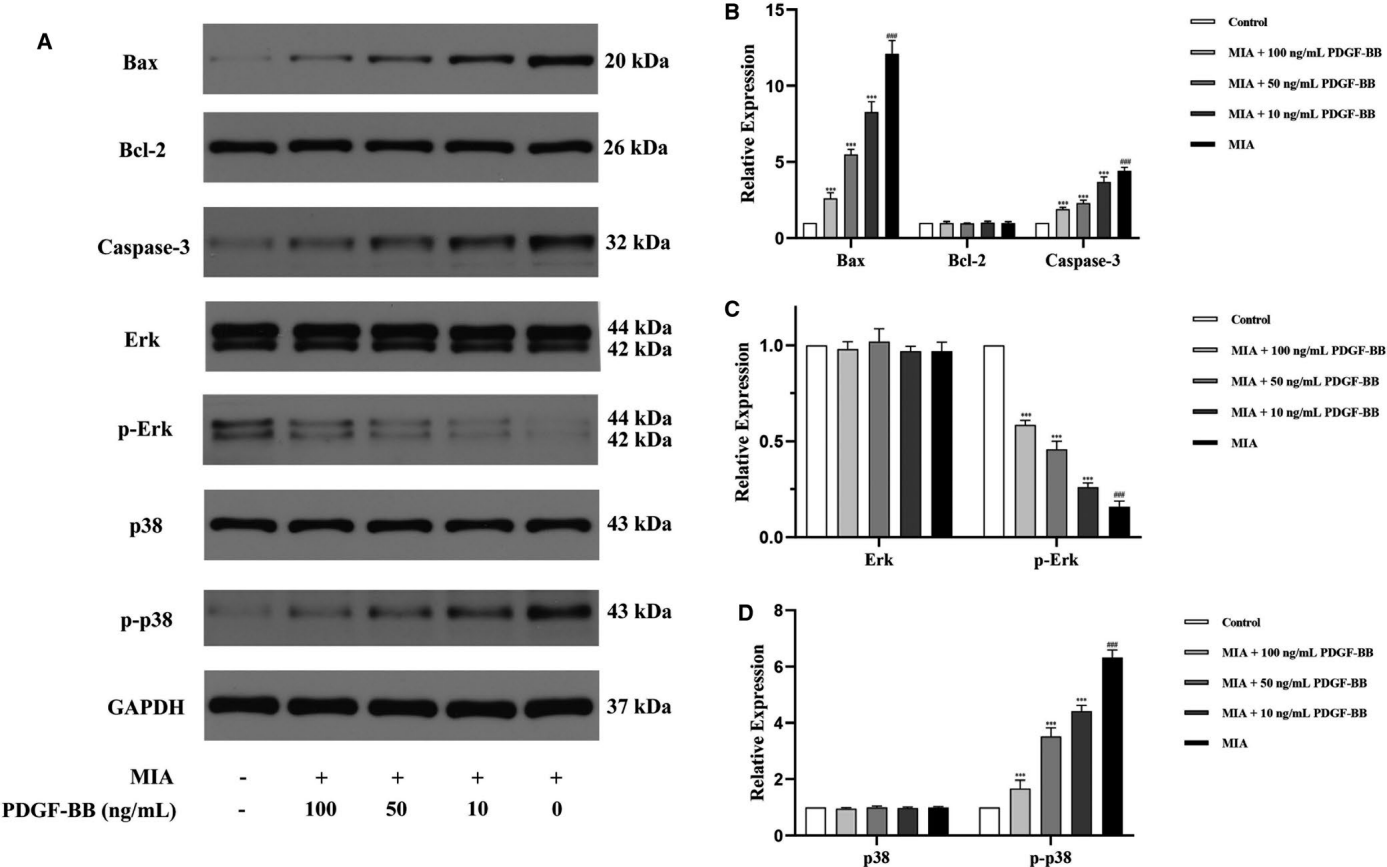
The effects of MIA and PDGF‐BB on Bax, Bcl‐2, caspase‐3, Erk, p‐Erk, p38 and p‐p38 in chondrocytes were assessed using Western blotting. (A and B) MIA up‐regulated Bax and caspase‐3 and showed no effects on Bcl‐2 in chondrocytes. The up‐regulated Bax and caspase‐3 were down‐regulated by PDGF‐BB. (A and C) MIA down‐regulated p‐Erk and showed no effects on Erk in chondrocytes. The down‐regulated Erk was up‐regulated by PDGF‐BB. (A and D) MIA up‐regulated p‐p38 and showed no effects on p38 in chondrocytes. The up‐regulated p38 was down‐regulated by PDGF‐BB. *, *P* < .05 vs. the MIA group; **, *P* < .01 vs. the MIA group; ***, *P* < .001 vs. the MIA group; and ###, *P* < .001 vs. the control group

**FIGURE 3:**
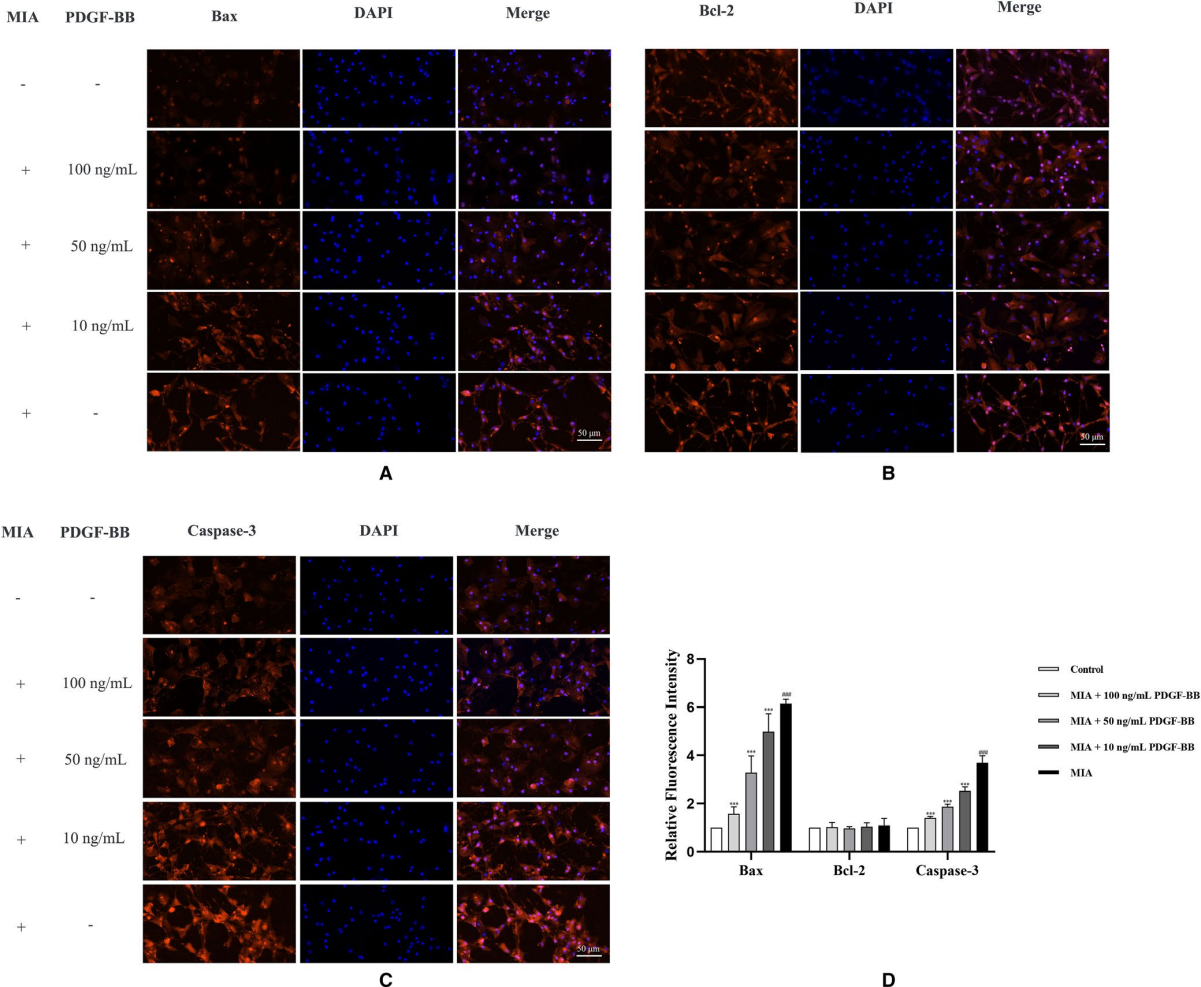
The effects of MIA and PDGF‐BB on Bax, Bcl‐2 and caspase‐3 were detected by immunofluorescence staining. (A and D) MIA up‐regulated Bax in chondrocytes. The up‐regulated Bax was down‐regulated by PDGF‐BB. (B and D) MIA and MIA+PDGF‐BB showed no effects on Bcl‐2 in chondrocytes. (C and D) MIA up‐regulated caspase‐3 in chondrocytes. Up‐regulated caspase‐3 was down‐regulated by PDGF‐BB

Bax, Bcl‐2 and caspase‐3, defined as apoptosis‐related factors, are increased in cell apoptosis and play a role in the progression of OA. We assessed the levels of apoptosis‐related factors in the MIA‐induced chondrocytes by Western blotting and immunofluorescence staining (Figures 2 and 3). Western blot results showed that the Bax and caspase‐3 proteins were present at higher levels in the MIA‐induced chondrocytes than in the normal chondrocytes. The results were consistent with the immunofluorescence analysis. We added different concentrations of PDGF‐BB to the MIA‐induced chondrocytes to confirm the antiapoptotic effect of PDGF‐BB. Both Western blot and immunofluorescence results demonstrated that PDGF‐BB could decrease the expression of Bax and caspase‐3. A series of experiments also demonstrated that a high dose of PDGF‐BB (100 ng/mL) presented a better antiapoptotic effect than a low dose of PDGF‐BB (10 ng/mL and 50 ng/mL).

**FIGURE 4:**
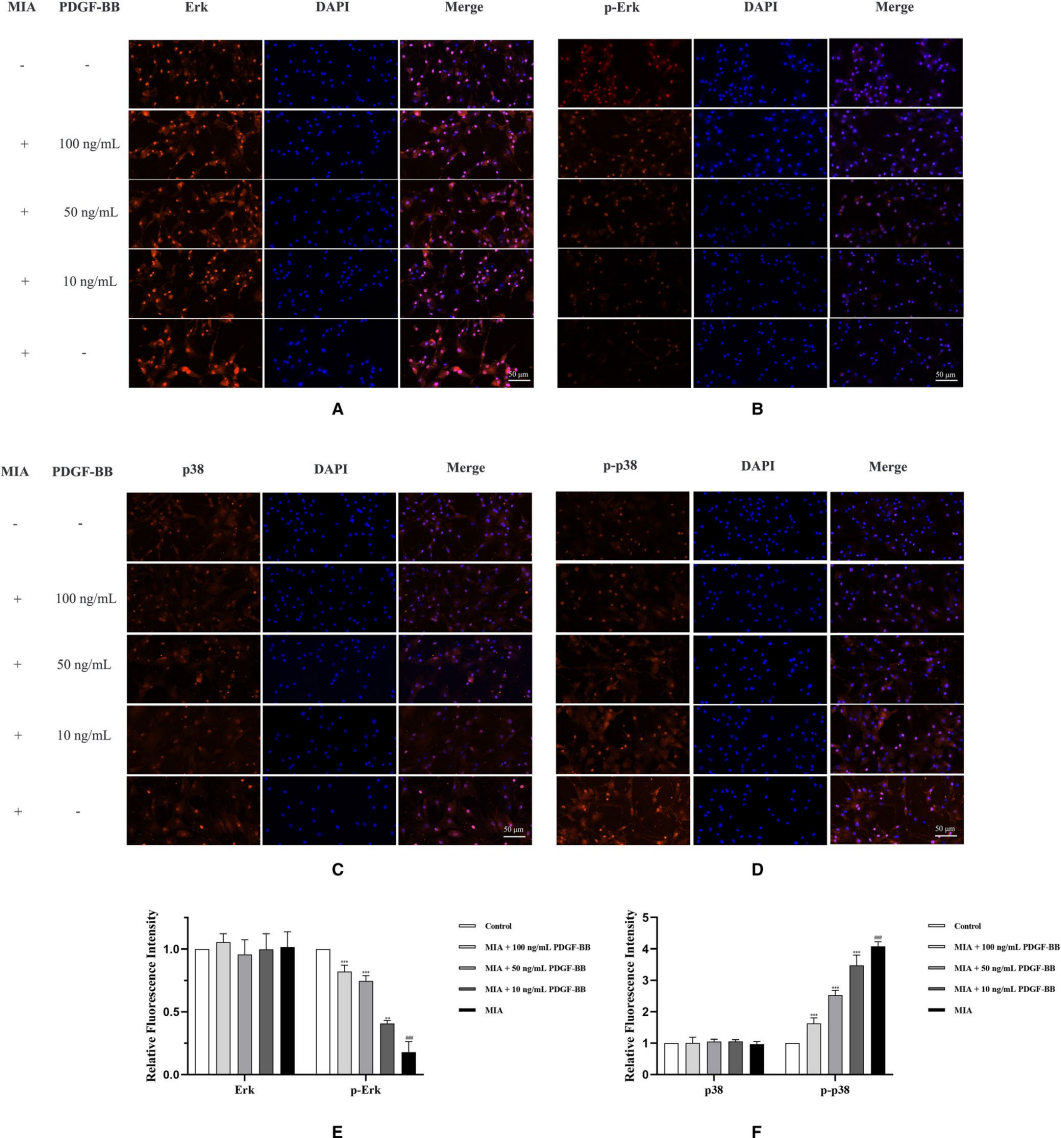
The effects of MIA and PDGF‐BB on Erk, p‐Erk, p38 and p‐p38 were detected by immunofluorescence staining. (A and E) MIA and MIA+PDGF‐BB showed no effects on Erk in chondrocytes. (B and E) MIA down‐regulated p‐Erk in chondrocytes. The down‐regulated p‐Erk was up‐regulated by PDGF‐BB. (C and F) MIA and MIA+PDGF‐BB showed no effects on p38 in chondrocytes. (D and F) MIA up‐regulated p‐p38 in chondrocytes. The up‐regulated p‐p38 was down‐regulated by PDGF‐BB. *, *P* < .05 vs. the MIA group; **, *P* < .01 vs. the MIA group; ***, *P* < .001 vs. the MIA group; and ###, *P* < .001 vs. the control group

Changes in the phosphorylation levels of Erk1/2 and p38 are closely related to the expression of Bax and caspase‐3, which in turn mediate chondrocyte apoptosis. In this study, Western blotting and immunofluorescence staining were used to determine whether Erk1/2 and p38 were differentially activated after PDGF‐BB treatment of the MIA‐induced chondrocytes (Figures 2 and 4). We first demonstrated that the MIA‐induced chondrocytes had a higher level of p38 and lower level of phosphorylated Erk. Western blot analysis revealed that Erk and p38 are phosphorylated in a dose‐dependent manner in the MIA‐induced chondrocytes after incubation with PDGF‐BB for 24 hours. Similarly, the immunoreactivity of p‐Erk was significantly increased in the 100 ng/mL PDGF‐BB group compared to the 10 ng/mL and 50 ng/mL groups. These data suggested that enhanced ERK phosphorylation and alleviated p38 expression participated in the antiapoptotic effect of PDGF‐BB in the MIA‐induced chondrocytes.

### 3.6 PDGF‐BB ameliorates the histopathology of OA in MIA‐induced cartilage degeneration osteophyte formation

**FIGURE 5:**
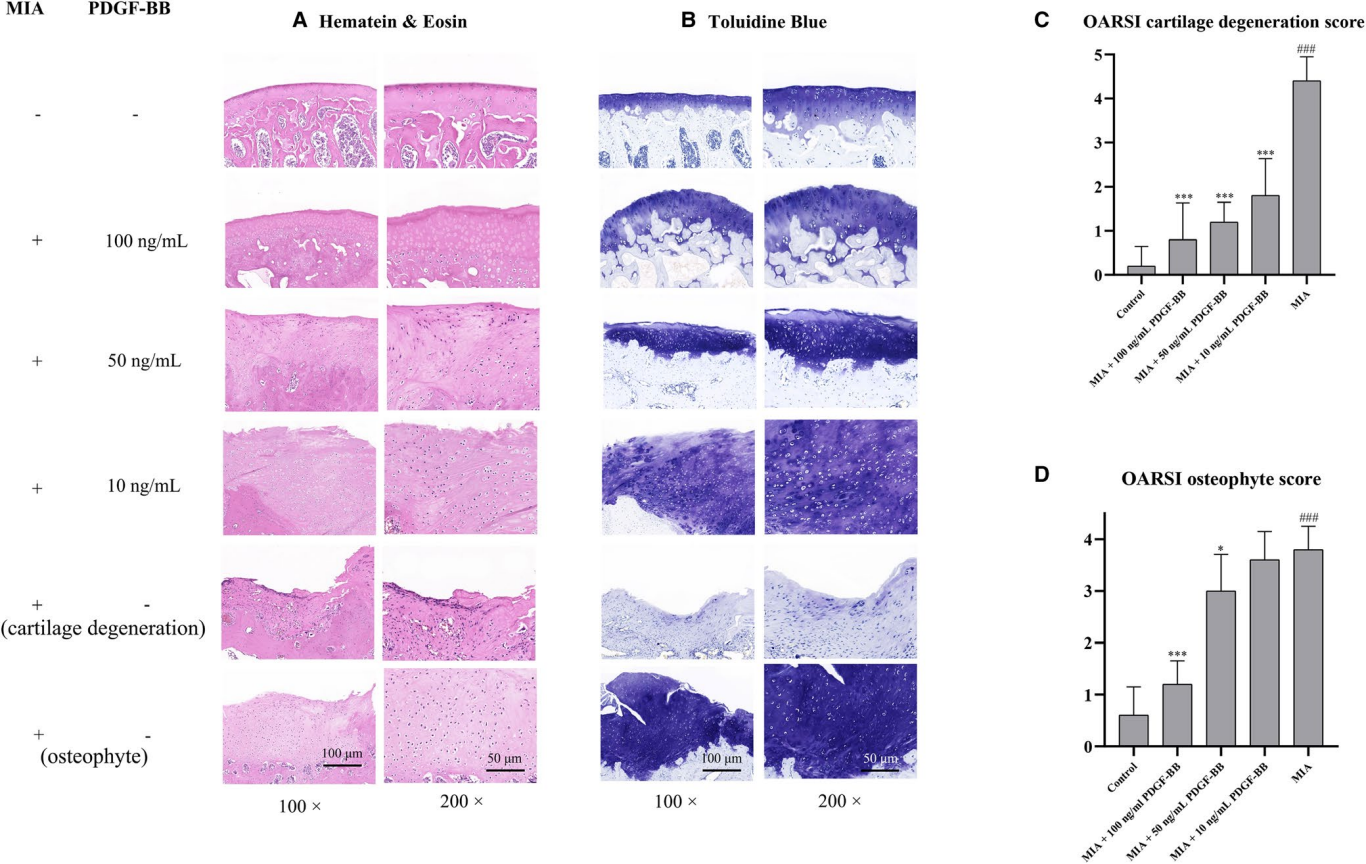
The histological features of the rat knee joint after treatment with MIA and MIA+PDGF‐BB. (A and B) The histological features were detected after HE staining and toluidine blue staining. In some areas, MIA leads to chondrocytes and matrix loss; in other areas, MIA leads to cartilage hyperplasia and large osteophyte formation. MIA+10 ng/mL PDGF‐BB reduced chondrocyte and matrix loss and showed little effect on cartilage hyperplasia. MIA+50 and 100 ng/mL PDGF‐BB reduced chondrocyte and matrix loss and alleviated cartilage hyperplasia and osteophyte formation. (C) Chondrocyte and matrix loss were evaluated using the OARSI cartilage degeneration score. MIA significantly increased the score. The increased score was decreased by 10, 50 and 100 ng/mL PDGF‐BB treatment. (D) Cartilage hyperplasia and osteophyte formation were evaluated using the OARSI osteophyte score. MIA significantly increased the score. The increased score was decreased by 50 and 100 ng/mL PDGF‐BB. *, *P* < .05 vs. the MIA group; **, *P* < .01 vs. the MIA group; ***, *P* < .001 vs. the MIA group; and ###, *P* < .001 vs. the control group

Histological changes demonstrated by HE and toluidine blue staining and the OARSI scores of cartilage tissues are shown in Figure 5. Histological analysis showed that the cartilage in the MIA group presented regional loss of matrix and chondrocytes and was almost replaced by scar tissues. Moreover, abnormal tissue hyperplasia and osteophyte formation were easily observed in the impaired areas. The PDGF‐BB groups presented significantly less matrix and chondrocyte loss than the MIA group; however, abnormal tissue hyperplasia was still observed in the 10 ng/mL and 50 ng/mL PDGF‐BB groups. In the 100 ng/mL PDGF‐BB group, there was less matrix and chondrocyte loss and almost no cartilage tissue hyperplasia. The OARSI scores of cartilage degeneration and osteophytes confirmed these observations. The OARSI scores were higher in the MIA group than in the PDGF‐BB group, in which the 100 ng/mL PDGF‐BB group showed the strongest changes and had the closest phenotype to that of normal cartilage.

### 3.7 PDGF‐BB increases the level of collagen II, decreases the levels of collagen X in both chondrocytes and matrix and decreases MIA‐induced apoptosis

**FIGURE 6:**
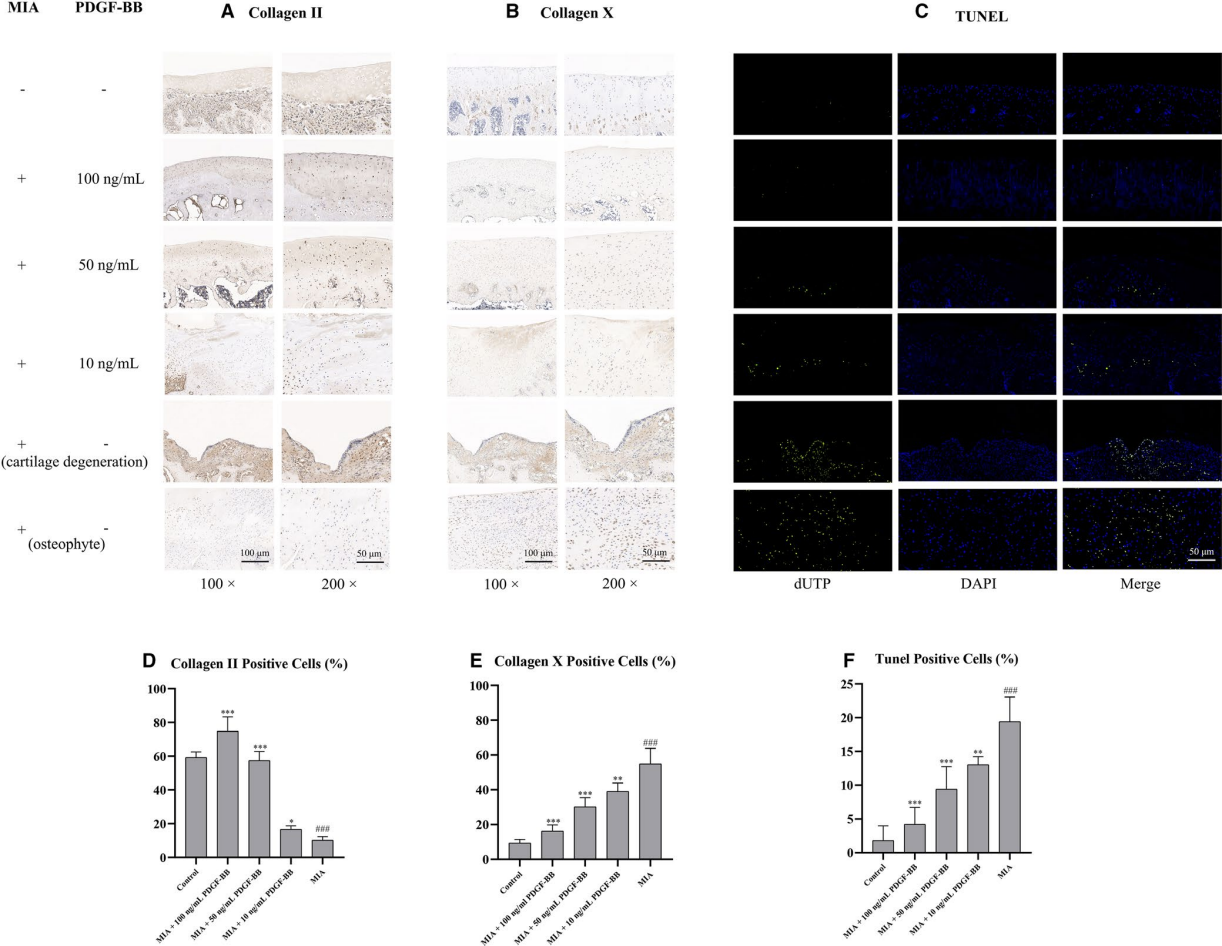
The effects of MIA and PDGF‐BB on the cartilage of rat knee joints. (A and D) The level of collagen II was detected by immunohistochemistry. MIA decreased the level of collagen II in both chondrocytes and matrix. The decreased level was increased by PDGF‐BB. The level of collagen II in the control group was similar to that in the MIA+50 ng/mL group. (B and E) The level of collagen X was detected by immunohistochemistry. MIA increased the level of collagen X in both chondrocytes and matrix. The increased level was decreased by PDGF‐BB. (C and F) The level of apoptosis in cartilage was detected by TUNEL staining. MIA increased the apoptosis rate in cartilage. The increased apoptosis was decreased by PDGF‐BB. *, *P* < .05 vs. the MIA group; **, *P* < .01 vs. the MIA group; ***, *P* < .001 vs. the MIA group; and ###, *P* < .001 vs. the control group

Collagen II and collagen X are important components in cartilage, and we next detected their contents in cartilage after MIA induction. As shown in Figure 6A,B,D and E, the immunochemistry results reflected the levels of collagen II and collagen X in cartilage tissues. In the MIA group, the levels of collagen II decreased and collagen X increased compared to those in the control group. In the PDGF‐BB‐treated cartilage tissue, the level of collagen II increased and the level of collagen X decreased with increasing PDGF‐BB concentrations. The levels of collagen II in the MIA group were similar to those in the 50 ng/mL PDGF‐BB group but were lower than those in the 100 ng/mL PDGF‐BB group. The level of apoptosis in cartilage tissue was measured by TUNEL assays (Figure 6C,F). The apoptosis rate was significantly increased in both the cartilage degeneration area and the osteophyte area in the MIA groups compared to the control group. In the PDGF‐BB groups, the apoptosis levels decreased with increasing PDGF‐BB concentration. These results indicated the antiapoptotic effect of PDGF‐BB on MIA‐induced cartilage.

## 4 DISCUSSION

Treatment options for OA, such as painkillers, anti‐inflammatory drugs and physiotherapy, remain focused on addressing pain rather than curing the disease. In our investigation of the effect of PDGF‐BB on OA, we found that it had protective effects on MIA‐induced OA models both in vitro and in vivo. As shown by the results described above, PDGF‐BB can attenuate OA via caspase‐3‐dependent apoptosis by regulating the phosphorylation of p38 and Erk in vitro. Moreover, the effects of PDGF‐BB on OA in vivo mainly include inhibiting chondrocyte loss, reducing cartilage hyperplasia and osteophyte formation, and regulating collagen anabolism, which contributes to remodelling cartilage tissue. Therefore, intra‐articular recombinant PDGF‐BB injection might be a potential conservative treatment for OA in humans.

Proliferation and apoptosis of chondrocytes, the only resident cells in cartilage, maintain a certain balance under normal circumstances. Apoptosis, as a highly regulated and active process of cell death, is critical for maintaining homeostasis. In the development of OA, the balance of these processes is disrupted, and the ratio of chondrocyte apoptosis is substantially increased. As early as 1998, Blanco et al observed a high proportion of apoptotic chondrocytes in osteoarthritic cartilage (51% vs. 11%).^19^ Subsequent studies also demonstrated that promoting apoptosis in chondrocytes could lead to OA.^20^, ^21^ Consistent with previous studies, we found that OA induced by MIA resulted in an increased apoptosis rate in chondrocytes. At present, the mainstream belief in the literature is that there is a correlation between the degree of cartilage damage and chondrocyte apoptosis. Therefore, preventing or curing OA by intervening in chondrocyte apoptosis would be a valid strategy to modulate cartilage degeneration. Wang et al and Ding et al found that inhibiting apoptosis could be a potential therapeutic strategy for OA treatment A previous report showed that intra‐articular injection of 10 ng/mL recombinant PDGF‐BB combined with hyaluronic acid could alleviate OA by promoting the anabolism of collagen II and inhibiting catabolism in rats.^16^ Based on the above description, PDGF‐BB promotes proliferation.^12^ We investigated whether PDGF‐BB has an antiapoptotic effect in OA. In this study, treatment with PFGF‐BB led to a decrease in the proportion of apoptosis in MIA‐induced OA, and a similar result was found in an in vitro study. Surprisingly, a different view of the effects of PDGF‐BB on OA has been reported. Sun et al found that an elevated serum level of endogenous PDGF‐BB contributed to the development of OA by stimulating angiogenesis.^22^ These researchers found that the concentration of serum PDGF‐BB is approximately 20 ng/mL,^22^ indicating that the concentration in the joint capsule should be much lower than that in the relatively poor blood supplement around the joints. As a result, the level of endogenous PDGF‐BB is too low to reverse the damage caused by OA.

Chondrocyte apoptosis in OA can occur via both caspase‐dependent (mainly caspase‐3 and caspase‐8) and caspase‐independent pathways.^23^ Phosphorylation of p38, as an upstream activator of the caspase‐3‐dependent pathway, plays an important role in the progression of OA.^24^ In addition, the alleviation of OA was closely correlated with the suppression of p38 activation.^25^ The phosphorylation of p38 could increase the Bax/Bcl‐2 ratio by up‐regulating Bax and/or down‐regulating Bcl‐2, resulting in caspase‐3‐dependent apoptosis.^26^, ^27^ In our research, we found that OA induced by MIA increased the phosphorylation of p38 and increased the Bax/Bcl‐2 ratio by up‐regulating Bax, contributing to caspase‐3‐dependent apoptosis. After treatment with PDGF‐BB, the phosphorylation of p38 decreased, resulting in down‐regulation of Bax and inhibition of caspase‐3‐dependent chondrocyte apoptosis. However, the activation of Erk is believed to be a negative factor for apoptosis and has been proven to be a protective factor in the progression of OA via apoptosis and autography.^28^, ^29^, ^30^, ^31^ In our research, the phosphorylation of Erk decreased after MIA treatment. After treatment with PDGF‐BB, the phosphorylation of Erk increased, accompanied by inhibition of chondrocyte apoptosis and improvement of OA. As a result, PDGF‐BB alleviated OA by suppressing apoptosis through down‐regulation of the p‐p38/Bax/caspase‐3 pathway and increasing Erk phosphorylation.

Numerous collagen subtypes, including collagen II, VI, IX, X, XII, XI and XIV, have been found in articular cartilage.^32^ Among these molecules, collagen II, which constitutes the main structure of collagen fibrils and provides tensile strength for the cartilage matrix, has the highest content.^33^ In contrast, collagen X constitutes only approximately 1% of the total collagen in articular cartilage.^34^ However, the production of collagen X increases in hypertrophic chondrocytes and hypertrophic cartilage.^35^, ^36^ Moreover, collagen X was found to be increased in osteoarthritic cartilage, especially in the vicinity of the lesion and hypertrophic zone, and was rarely detected in normal chondrocytes.^37^, ^38^ Unlike collagen II, collagen X is believed to be a type of network‐forming collagen that can maintain tissue stiffness, regulate chondrocyte metabolism and interact with hypertrophic chondrocytes.^32^, ^39^ In our results, PDGF‐BB up‐regulated collagen II and down‐regulated collagen X in chondrocytes and cartilage in vivo. Furthermore, collagen X decreased along with the improvement in cartilage hypertrophy and osteophyte formation. This finding demonstrated that PDGF‐BB could reverse matrix loss and inhibit cartilage hyperplasia caused by OA. As a result, an appropriate concentration of PDGF‐BB could alleviate OA and remodel cartilage tissue by inhibiting chondrocyte loss and reducing cartilage hyperplasia and osteophyte formation.

OA is primarily characterized by cartilage degradation, and cartilage hyperplasia and osteophytes are also important features.^40^, ^41^, ^42^ Hashimoto et al found that almost all cells in the hypertrophic cartilage zone and some cells in the proliferative zone underwent apoptosis 9 and 12 weeks after induction of OA in rabbits, as shown by TUNEL staining.^40^ In the MIA‐induced OA model in our study, both severe cartilage degradation and hyperplasia occurred, and the rate of apoptosis was also significantly higher than that in normal cartilage. After treatment with 10 ng/mL PDGF‐BB, cartilage degradation was apparently improved. However, cartilage hyperplasia still existed and was not notably improved. After treatment with 50 ng/mL PDGF‐BB, cartilage hyperplasia was significantly alleviated, while large osteophytes could still be observed. After treatment with 100 ng/mL PDGF‐BB, cartilage hyperplasia and osteophyte formation almost disappeared. As shown by TUNEL assays, the apoptosis rate decreased along with the increase in PDGF‐BB concentration, the improvement in cartilage degradation, the alleviation of cartilage hyperplasia and the lack of osteophyte formation, consistent with the results of Hashimoto et al.^40^

In the pathogenesis of OA, NO and PGE2 are typical inflammatory markers and play important roles. NO is a common inflammatory mediator that can cause a decrease in collagen II and proteoglycan synthesis, leading to matrix loss.^43^ Moreover, NO is believed to induce chondrocyte apoptosis, leading to cartilage degeneration.^23^ PGE2 is another inflammatory mediator that can cause extracellular matrix degradation and inhibit chondrocyte proliferation.^44^ Studies have shown that inhibiting the production of inflammatory mediators such as NO and PGE2 could attenuate the progression of OA.^45^, ^46^ In our study, we found that PDGF‐BB could down‐regulate these inflammatory mediators, indicating that PDGF‐BB exhibited anti‐inflammatory effects and could be a potential therapy for OA.

A systematic review and meta‐analysis proved that intra‐articular platelet‐rich plasma (PRP) injection could reduce pain and improve joint functions in OA.^47^ The greatest advantage of PRP is its low immunogenicity because it is derived from autologous blood. However, the greatest advantage is also its greatest limitation because of the high demand with regard to the operation and equipment. When patients receive PRP treatment, their blood will be drawn first and centrifuged or apheresis will be used to gather the platelets, and then, PRP is injected into their own joints. It is difficult for PRP to be repeatedly administered and developed as a commercial drug for easy application. In addition, this treatment is not suitable for patients with anaemia and platelet dysfunction. PFGF‐BB is one of the effective factors in PRP.^48^ The concentration of PDGF‐BB in centrifuge‐prepared PRP is approximately 10 ng/mL, which is close to the minimum dose we used in this research.^47^ For the frequency of injection, we applied PRP once a week for 4 weeks, consistent with our previous clinical research on PRP for treatment of musculoskeletal disease.^49^ According to the results above, high concentrations of PDGF‐BB and/or other effective growth factors in platelets may replace PRP and be developed as commercial treatments for easy application. However, further analyses should be conducted to compare the safety and effects.

This research still has limitations. First, we found that PDGF‐BB alleviates OA via caspase‐3‐dependent apoptosis by regulating the phosphorylation of p38 and Erk. Further mechanisms of this effect and crosstalk among signalling pathways were not discovered. Second, PRP has been proven effective in OA, and although a high concentration of recombinant PDGF‐BB could be used to overcome the limitations of PRP, the effects of recombinant PDGF‐BB and PRP should be further compared.

**FIGURE 7:**
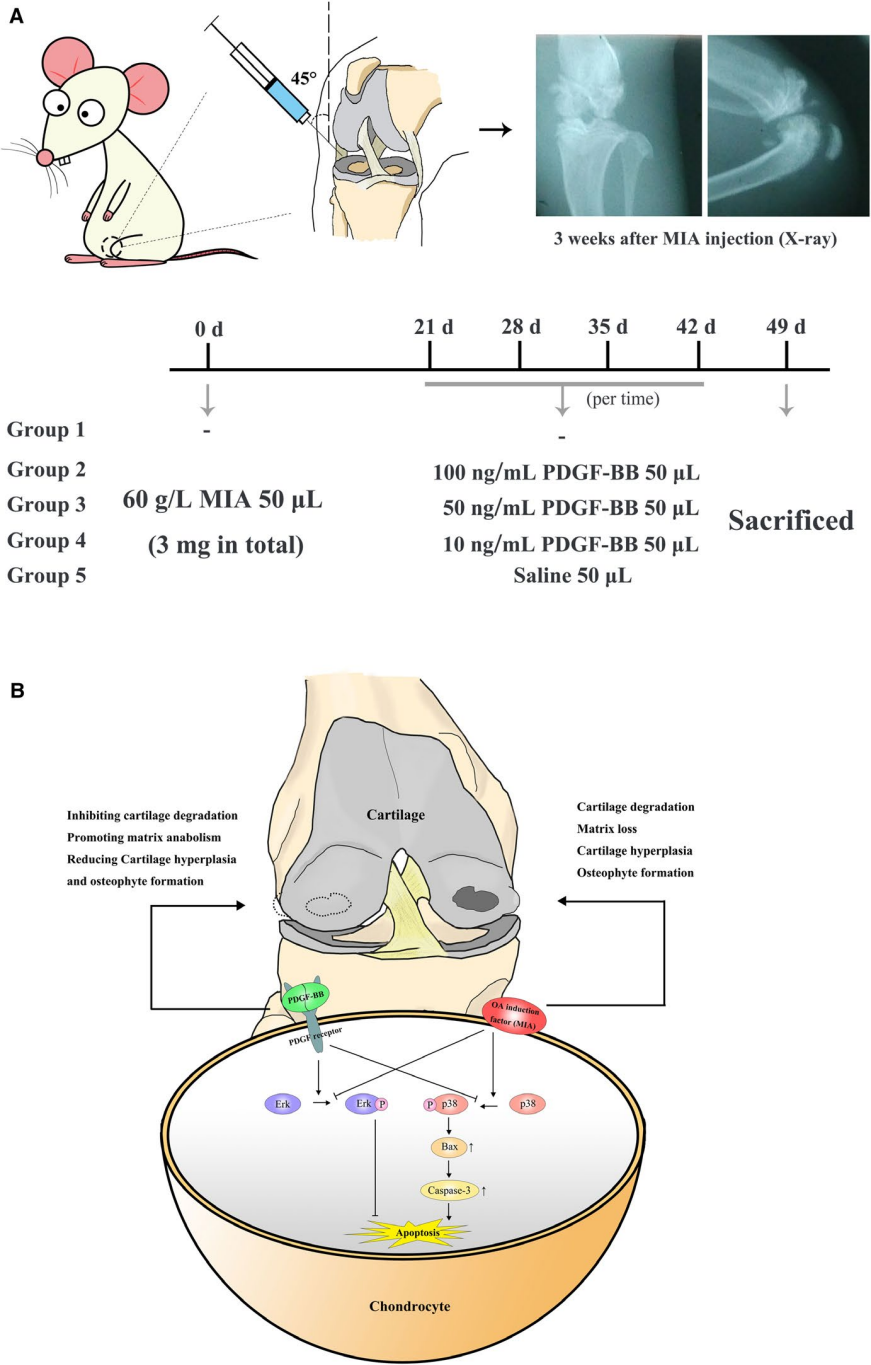
A, The procedure for animal experiments, including both OA modelling and PDGF‐BB intervention in rats, in our research. B, Schematic model of the effects of MIA and PDGF‐BB on OA at both cellular and tissue levels

In conclusion, as shown in Figure 7B, recombinant PDGF‐BB alleviated OA induced by MIA in a rat model by down‐regulating caspase‐3‐dependent apoptosis. The decrease in Bax and phosphorylation of p38 and the increase in phosphorylation of Erk are involved in this process. The effects of PDGF‐BB on osteoarthritic cartilage mainly include inhibiting chondrocyte loss, reducing cartilage hyperplasia and osteophyte formation, and regulating collagen anabolism, which contribute to remodelling cartilage tissue.

## CONFLICT OF INTEREST

The authors declare that they have no conflict of interest.

## AUTHOR CONTRIBUTIONS

**Pengfei Zhu:** Investigation (equal); Methodology (equal); Project administration (equal); Writing‐original draft (equal); Writing‐review & editing (equal). **Zhengchao Wang:** Investigation (equal); Methodology (equal); Project administration (equal); Writing‐original draft (equal); Writing‐review & editing (equal). **Zhenxing Sun:** Investigation (equal); Methodology (equal); Project administration (equal); Writing‐original draft (equal); Writing‐review & editing (equal). **Bokai Liao:** Investigation (equal); Writing‐review & editing (equal). **Yu Cai:** Funding acquisition (equal); Investigation (equal); Methodology (equal); Validation (equal); Writing‐original draft (equal).

## ETHICAL APPROVAL

All experimental animals were maintained in accordance with the Guide for the Care and Use of Laboratory Animals of the National Institutes of Health and were approved by the Ethics Committee for Animal Experimentation of Puai Hospital Affiliated with Huazhong University of Science and Technology.

## Data Availability

The data support the findings of this study are available from the corresponding author upon reasonable request.
